# Study of Corrosion, Structural, and Mechanical Properties of EN AW-6082 and EN AW-7075 Welded Joints

**DOI:** 10.3390/ma14164349

**Published:** 2021-08-04

**Authors:** Tomasz Wojdat, Paweł Kustroń, Karol Jaśkiewicz, Jarosław Pabian

**Affiliations:** 1 Department of Metal Forming, Welding and Metrology, Faculty of Mechanical Engineering, Wroclaw University of Science and Technology, 50-370 Wrocław, Poland; pawel.kustron@pwr.edu.pl (P.K.); karol.jaskiewicz@pwr.edu.pl (K.J.); 2 Mahle Behr Ostrów Wielkopolski Sp. z o.o., 63-400 Ostrów Wielkopolski, Poland; jaroslaw.pabian@mahle.com

**Keywords:** aluminum welding, hot cracks, CMT method, EN AW-7075 alloy, aluminum corrosion

## Abstract

The purpose of the work was to test the welded joints of aluminum alloys EN AW-7075 and EN AW-6082, which are used to join individual structural elements of car bodies, e.g., B-pillar with the body. The joints were made using the low-energy cold metal transfer (CMT) arc welding method. The results of the structure investigations of lap and butt joints, as well as tests of mechanical properties are presented. The influence of linear energy and the way of arranging materials in lap joints on the possibility of hot cracks occurrence has been demonstrated. The shear strength of lap joints was equal to 150 MPa, while the tensile strength of butt joints was equal to 375 MPa. The highest hardness reduction was observed in the heat affected zone (HAZ) from the EN AW-7075 alloy side in the range of 98 to 138 HV 0.05. In addition, a significant reduction of the corrosion resistance in the transition zone between HAZ and the base material (EN AW-7075 alloy) in the medium salinity environment, corresponding to the sea conditions according to ASTM G85 was indicated.

## 1. Introduction

The application of aluminum alloy sheets in the construction of car bodies is now more and more common. This is, not only European, but also a global trend caused by newly introduced directives and pro-ecological regulations, including, e.g., reduction of CO_2_ emissions [1] and other contaminants (HC, NOx, PM, and particulate matter) coming from the combustion of fuels in gasoline and diesel engines [2]. The reduction of the vehicle’s total weight using lightweight metal alloys for its construction is one of the key factors having a direct impact on the reduction of exhaust gases emissions. Currently, leading automotive companies offer cars, mainly a premium class, whose body and sheathing are made almost entirely of aluminum alloys. Replacing some components made of high-strength steel grades, e.g., boron-manganese steels, which are designed to provide high rigidity and strength of constructed elements, on light metal alloys, is a great technological challenge. As, for example, the B-pillar which rather than steel, can also be made of high-strength aluminum alloy EN AW-7075 [3]. Applying such a solution, however, causes considerable difficulties in assembly technology, especially in welding methods. This alloy has very limited weldability, which excludes the possibility of using conventional welding methods [4].

The weldability of the aluminum 7xxx series alloys is limited by the alloying additive of Cu in an amount exceeding 1 wt %. It causes hot cracks in welds or heat affected zone (HAZ) as a result of melting the metal and mixing it with the filler metal during the welding process, using conventional methods such as metal inert gas (MIG) or Tungsten inert gas (TIG) [5,6,7]. These methods are carried out with high linear energy, which affects the relatively high degree of mixing of metals, thus increasing the risk of hot cracks. Moreover, the high-energy course of the process causes the formation of a dendritic structure in HAZ, which leads to a significant reduction in the mechanical properties and corrosion resistance of these alloys [8]. Modern methods of laser beam welding or hybrid laser/gas metal arc (GMA) welding also have a significant limitation in this case. The reason is the high-power density of the integrated laser beam, compared to arc welding methods, increasing the risk of hot cracks, and causing intense metal evaporation, which in turn generates many gas pores [9]. Joints of good mechanical properties and high quality of aluminum 7xxx alloys can be obtained using the friction welding process, in particular friction stir welding (FSW) [10,11,12]. Furthermore, Umar et al. [13] describes that the FSW process makes it possible to obtain good quality joints not only EN AW 6082 aluminum alloy sheets, but also cast of AA6082/ZrO/BC composites. There are also joining attempts with the use of low-energy welding methods, such as cold metal transfer (CMT) [14,15].

In the study, the low-energy CMT method was used to produce welded joints. This is a modification of the GMA welding process, which significantly reduces the amount of heat input to the welding zone. This is achieved as a result of metal transfer in a short arc using the reciprocating wire feeding (RWF) technology [16,17]. The heat input amount controlled by selecting the appropriate welding parameters makes it possible to obtain welded joints of aluminium EN AW-7075 alloy without hot cracks [14].

## 2. Materials and Test Methodology

In the study, car body sheets made of aluminum EN AW-6082 and EN AW-7075 alloys were welded with the use of S Al 5183 (AlMg4.5Mn0.7) filler metal. The welding process was carried out on a robotic setup using the CMT method. A welding power source from Fronius, model TransPuls Synergic 3200 CMT (Fronius GmbH, Upper Austria, Wels, Austria) was used. The tests were carried out in a similar way to those described in [14], with the difference of filler metal with better mechanical properties used to make the joints. The first trial was in the following configuration: EN AW-7075 up and EN AW-6082 down. Secondly, the order was reversed. Wojdat et al. [14] indicated that a different materials configuration has strongly influenced the hot cracks occurrence in the lap joints. With properly selected parameters of the CMT process that provide low linear energy, hot cracks did not occur when the EN AW-7075 alloy is melted from the front surface, i.e., in butt and lap joints in which it is in the “up” position. In the reverse configuration, cracks do not occur only for the minimum parameters ensuring process stability [14]. The chemical composition of the base materials, measured by X-ray fluorescence analyzer Fischerscope X-ray XDL-B (Fischer GmbH, Achern, Badenia-Wirtembergia, Germany), was presented in Table 1 compared with the range of element concentrations acc. to the standards [18,19].

The joints were produced on a robotized setup in order to obtain repeatable welding conditions and to make joints for very similar process parameters to those described in [14]. The welding speed was constant and was equal to 100 cm/min. The other parameters changed depending on the wire feed speed, which was changed in the range of 6.5 to 7.5 m/min. Therefore, the current was in the range of 140–165 A, and the arc voltage was 15.1–16.3 V. The welding linear energy (the heat input) was equal to 1.0–1.3 kJ/mm. The welding parameters were according to the synergic curve of CMT power source. The pure argon 4.5 (99.995% Ar) was used as the shielding gas.

**Figure 1:**
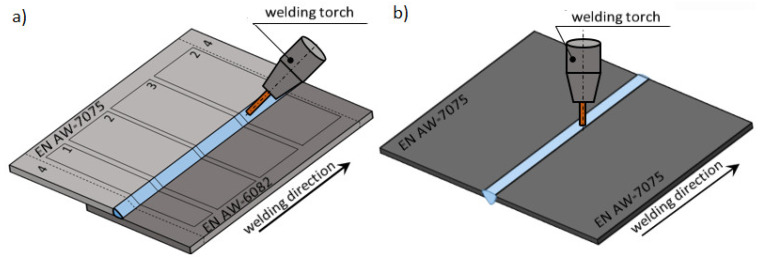
Method of welding lap joints (**a**) and butt joints (**b**) along with the method of sampling for testing (**a**): 1. Metallographic tests, 2. tests of mechanical properties (static shear test), 3. additional samples, 4. waste (25 mm).

The joints for the tests were made by welding together aluminum sheets with a thickness of 3 mm and dimensions of 100 × 250 mm arranged with an overlap of 10 mm along the longer side. The welding torch was positioned at 45° (Figure 1a) for lap joints and 90° for butt joints (Figure 1b). Subsequently, samples were cut from them for microscopic and mechanical properties studies (Figure 1a). The same set of samples were made for corrosion resistance tests. As was shown in [14], in the case of butt joints, the fracture that occurs in the base material of lower strength (EN AW-6082 alloy) at the average tensile strength was equal to 258 MPa. For this reason, the butt joints were made by joining two sheets of the EN AW-7075 alloy together. The aim was to determine the fracture mechanism and tensile strength of joints made using filler metal with higher mechanical strength.

The structure of the welded joints was assessed using light microscopy (LM) and scanning electron microscopy (SEM). Samples cut out from lap joints were subjected to a static shear test, while samples from butt joints to a static tensile test, and then the resulting fractures were rated. In addition, hardness measurements were performed using the Vickers method in individual zones of welded joints, including: Base material (BS), heat affected zone (HAZ), heat zone (HZ), and the weld. All of the studies were performed in accordance with the applicable standards [20,21,22,23].

The influence of the welding process on the corrosion resistance of welded joints of aluminum sheets EN AW-6082 and EN AW-7075 was tested in the ASTM G85 appendix A3-SWAAT test (sea water acetic acid test). The SWAAT test is a corrosion test that uses synthetic sea salt rather than traditional sodium chloride. The solution is then acidified with glacial acetic acid. It is carried out in the conditions of the so-called wet bottom. The pH of the saline solution should be in the range 2.8–3.0. The temperature of the saturator tower (bubble tower) should be kept at 47 ± 1 °C if the salt chamber temperature is 35 °C and 57 ± 1 °C if the temperature in the salt chamber is 49 °C. The SWAAT test cycle is 30 min of spraying followed by 90 min of soaking at 98% relative humidity (RH). The duration of the SWAAT test was 20 days. The samples were prepared in the same way as shown in Figure 1. The SWAAT test was carried out using the Corrosionbox 400e (Cofomegra, Milano, Italy).

## 3. Results and Discussion

### 3.1. Metallographic Tests

**Figure 2:**
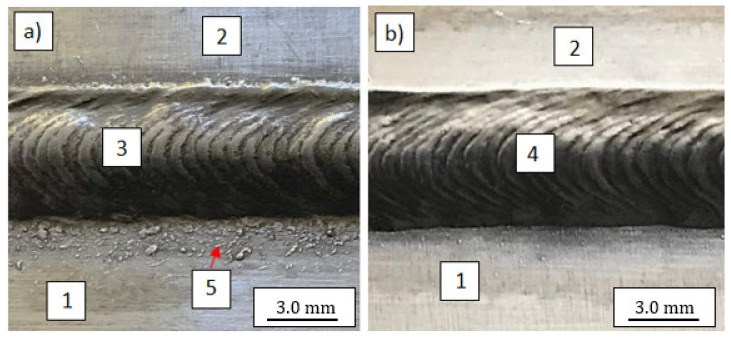
The lap joints welded by different filler metals: S Al 5183 (**a**) and S Al 4047 (**b**): 1. EN AW-7075 alloy, 2. EN AW-6082 alloy, 3. S Al 5183 weld, 4. S Al 4047 weld, 5. spatters.

The low magnification observations of samples was done by scanning light microscope Keyence VHX1000 (Keyence, Osaka, Japan). The visual tests carried out after the welding process showed a significant amount of small spatters in the welding zone very close to the weld (Figure 2a). Such imperfections did not occur during welding with the use of S Al 4047 filler metal (Figure 2b). The study of the structure of similar joints, but welded using different filler metals (S Al 4047), was described in [14] by Wojdat et al.

**Figure 3:**
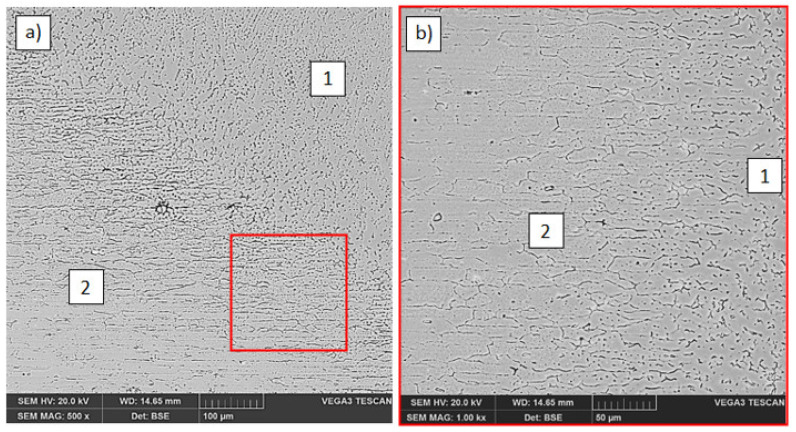
Fusion line between the weld and EN AW-7075 alloy melted from the front surface in the lap joint (**a**) and significant grain growth in the HAZ of EN AW-7075 alloy (**b**): 1. weld, 2. HAZ from EN AW-7075 alloy side.

The microstructural studies by scanning electron microscopy were carried out on a Tescan Vega3 (Tescan, Brno-Kohoutovice, Czech Republic). The observations were done under back scattered electrons (BSE). In the microstructure of butt and lap joints, in which the EN AW-7075 alloy was in the up position and was melted from the front surface (Figure 3a), changes in the structure (significant grain growth) in the HAZ are visible (Figure 3b).

**Figure 4:**
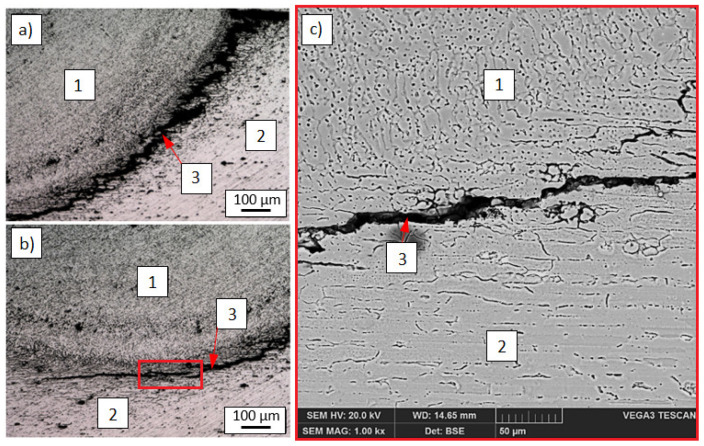
Hot cracks in welded lap joint—hot cracks spreading from the sheet metal edge along the fusion line: Light microscopy (**a**,**b**) and SEM (**c**): 1. weld, 2. aluminum EN AW-7075 alloy, 3. hot cracks.

However, significant differences could be seen in the structure of lap joints made in the reverse configuration. Regardless of the welding parameters, the joints exhibited hot cracks spreading from the sheet metal edge (EN AW-7075 alloy) along the fusion line (Figure 4). As a result, welding of the EN AW-7075 alloy using the S Al 5183 filler metal is only possible when it is melted from the front surface.

**Figure 5:**
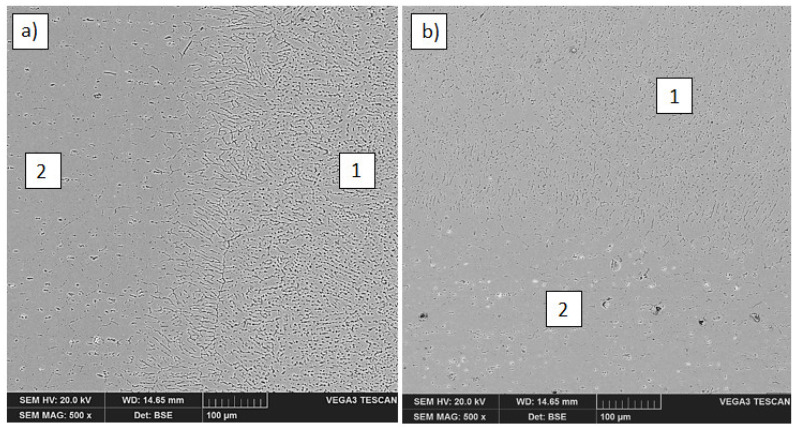
Structure of HAZ in the lap joint from the EN AW-6082 alloy side melted from the front surface (**a**) and the reverse configuration joint (**b**): 1. weld, 2. EN AW-6082 alloy.

From the EN AW-6082 alloy side, regardless of whether it was melted from the front surface (butt joint and the lap joint in which it was in the up position of the joint (Figure 5a)) or in reverse configuration in which it was at the bottom of the lap joint (Figure 5b), no clear changes in the structure were observed in HAZ. The joint shows a distinct fusion line in the transition zone between the weld and the EN AW-6082 alloy.

**Figure 6:**
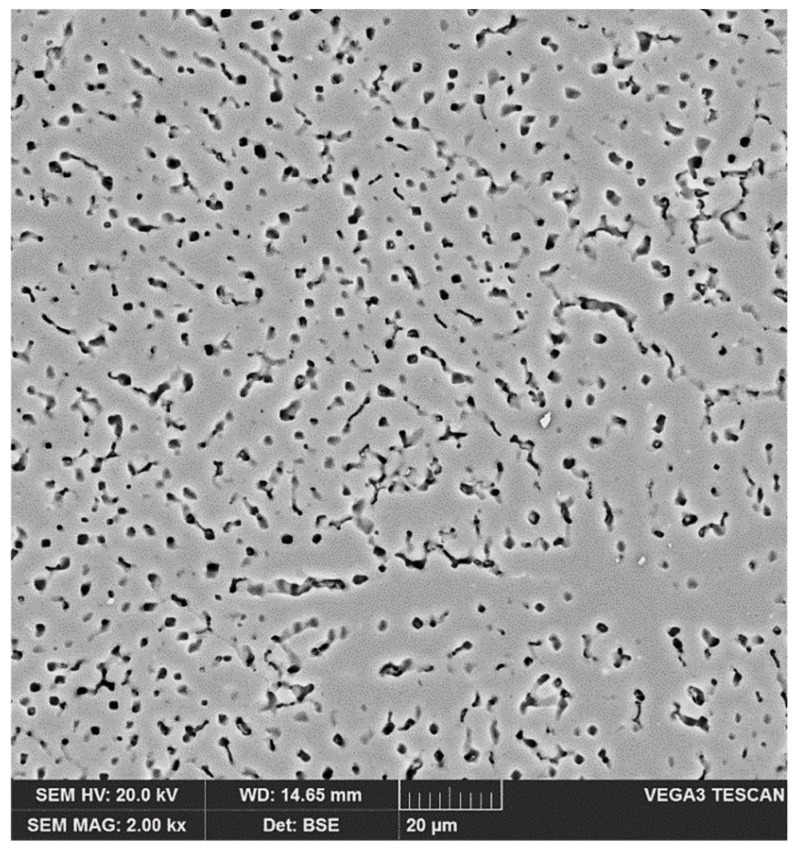
The back scattered electrons (BSE) image of weld-solid solution *α* (light background) and precipitation of the *β* phase (dark points).

The weld structure (in the concentration range up to approx. 35 wt % Mg [24]) is an eutectic mixture of the *α* solution with the *β* secondary solid solution (Figure 6). The microstructure of the weld thus consists of a solid solution *α* (light background) and precipitation of the *β* phase (dark points). On the back scattered electrons (BSE) image it can be observed that the *β* phase precipitates arise at the grain boundaries of the *α* solid solution.

### 3.2. Tests of Mechanical Properties

**Figure 7:**
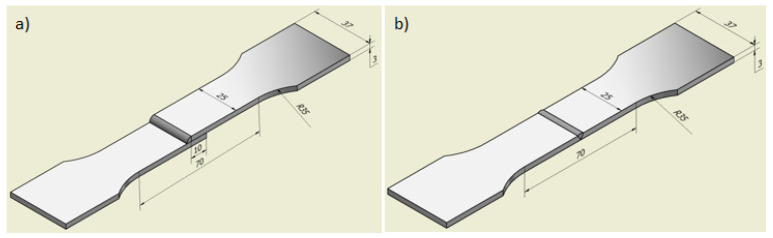
Shape and dimensions of samples for: (**a**) Static shear (lap joints) and (**b**) tensile (butt joints) tests.

The static shear test of lap joints and the static tensile test of butt joints were carried out on a Zwick/Roell Zmart-PRO testing machine (Zwick-Roell GmbH, Baden-Württemberg, Ulm, Germany). The samples for tests were prepared in accordance with the guidelines of the standard [21]. They were 25 mm wide in the measuring part and 37 mm in the grip part (Figure 7). Furthermore, The lap joints were positioned in the machine grips with the use of suitable distance inserts, due to the non-axial state of stress. The stretching speed was equal to 2 mm/min.

The results of the static shear test of dissimilar lap joints of EN AW-6082 and EN AW-7075 alloy with two different configurations and the results of static tensile test of butt joints, which was made by joining two sheets of the EN AW-7075 alloy together, are shown in Table 2. For each type of joint, six sets of samples were prepared.

**Figure 8:**
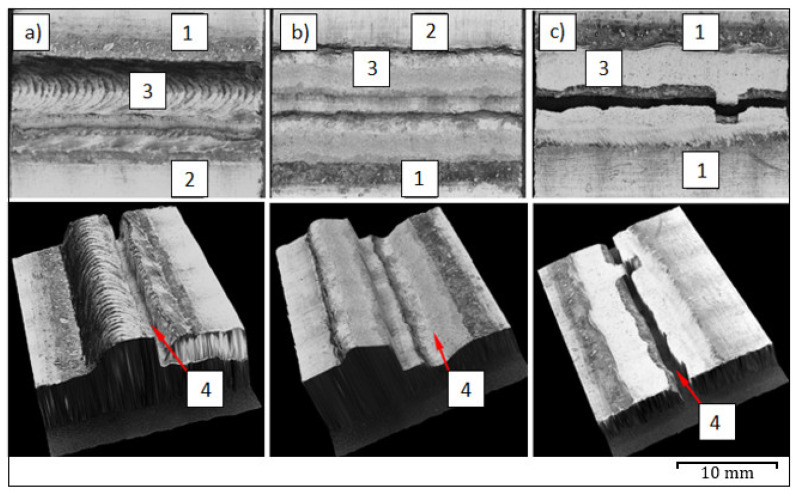
Examples of fracture of welded joints: Aluminium EN AW-7075 alloy in the up position of lap joint (**a**), reverse configuration of lap joint (**b**), butt joint (**c**); 1. EN AW-6082 alloy, 2. EN AW-7075 alloy, 3. weld, 4. fracture.

The mechanism of joint destruction of the lap joints in the static shear test for joints, in which the EN AW-7075 alloy was in the up position, occurred in the joint right next to the fusion line into the base material (Figure 8a). The average shear strength of such joints was above 150 MPa. As described in [14], the shear strength of analogous joints made with the use of a different filler metal (S Al 4047) is lower by approx. 40% and amounts to an average of 110 MPa.

For the reverse configuration of materials, the destruction of the joints that occurred as a result of the extraction of the weld along the fusion line from the EN AW-7075 alloy side-the weld, was torn out from the base metal (Figure 8b). Such a mechanism of joint destruction was initiated by a crack spreading from the sheet metal edge along the fusion line in the EN AW-7075 alloy. Therefore, the strength of the joints was lower and was equal to 105 MPa on average.

As shown in [14], the destruction of butt joints occurred in the base material with lower mechanical properties (EN AW-6082 alloy), and the average tensile strength was equal to 258 MPa. Therefore, a similar result could be expected if the joints were made using a higher strength filler metal (S Al 5183). For this reason, butt joints were made only for the EN AW-7075 alloy. In this case, the fracture was located in the weld right next to the fusion line into the base material (Figure 8c), and the average tensile strength was equal to 375 MPa. The much lower strength of lap joints results from the complex state of stresses occurring on the joint during shear force in tension, where apart from the shear forces there is also a bending moment.

Bolewski et al. [7] presents the results of stretching of welded joints made using the MIG-Puls method and the S Al 4043 filler metal. The strength of such joints was on average 250 MPa, and the fracture mechanism was located in the weld. Pfeifer et al. [15] described that the tensile strength of EN AW-7075 butt welded joints made using the CMT method and the S Al 2319 (AlCu6MnZrTi) filler metal was in the range of 285–305 MPa. The fracture mechanism was located in the weld, as well.

Microhardness measurements of welded joints were made using the Vickers method, according to the EN ISO 6507-1 standard [22], using a Micro Vickers Hardness Tester HVS-1000 from Sinwon (Sinowon, DongGuan City, Guangdong Province, China). The Vickers microhardness was also measured in individual zones of the welded joints (base material (BS), heat affected zone (HAZ), heat zone (HZ), and weld) five measurements were made in each zone. Due to the small width of HAZ and HZ, the load of indenter was equal 0.49 N. The results of Vickers microhardness measurements are shown in Table 3.

The highest hardness, regardless of the type of joint, was found in the EN AW-7075 aluminum alloy and was equal to 138.3 HV 0.05 on average. In HAZ, on the side of the EN AW-7075 alloy, there was a significant reduction in hardness, which was in the range of 98–128 HV 0.05, decreasing towards HZ. The reasons for the significant reduction in hardness in HAZ are changes in the structure (significant grain growth), which were demonstrated in metallographic tests. Hardness in the weld was equal to 105.7 HV 0.05 on average. In the transition zone from the side of the EN AW-6082 alloy, the hardness was similar to the hardness of the EN AW-6082 alloy, where it was equal to 80.6 HV 0.05 on average. The values presented above are average values obtained from measurements in all welded joints.

As shown in [14], in joints made with the use of S Al 4047 filler metal, the distribution of hardness in the individual zones of the joint is very similar. The difference is in the hardness of the weld, the average hardness which is 94 HV 0.05. The results are presented in [8], where the HAZ hardness in the EN AW-7075 alloy is in the range of 110–120 HV. A significant difference occurs in the base material, in which it is in the range of 180–190 HV and results from a different degree of hardening of the EN AW-7075 alloy. In these tests, the EN AW-7075 alloy was welded in the T4 hardened state, and in [8] the T6 hardened state.

### 3.3. Corrosion Tests

**Figure 9:**
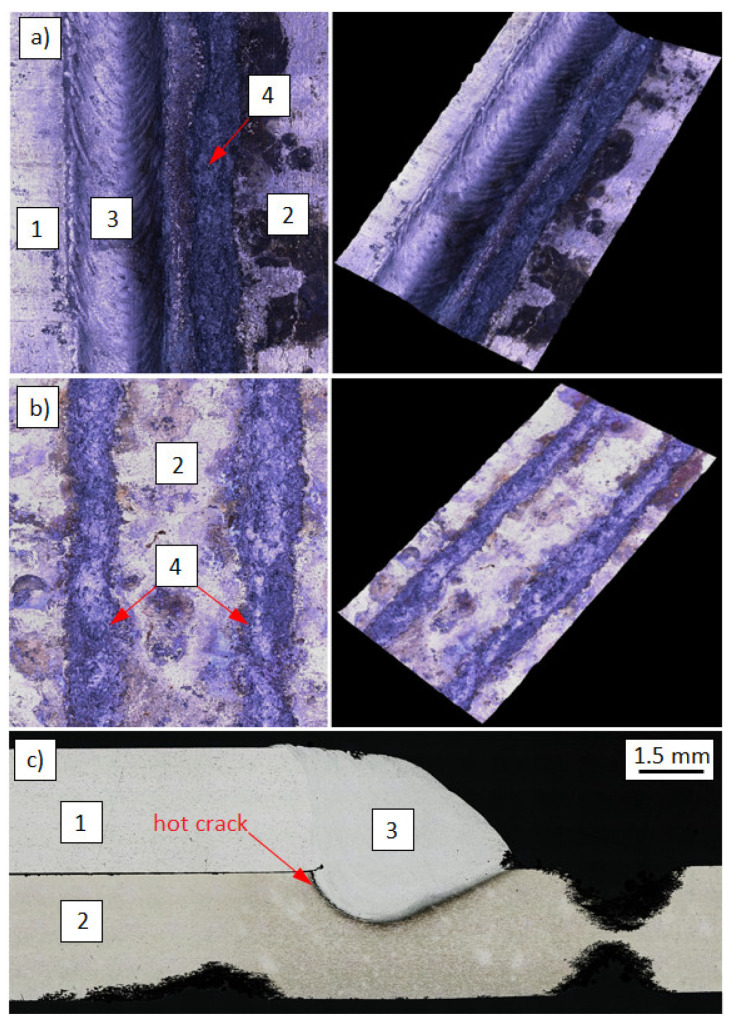
Lap welded joint after the corrosion SWAAT test where the EN AW-7075 alloy was stacked on the bottom-view of weld face side (**a**), back side (**b**), and cross section (**c**): 1. EN AW-6082 alloy, 2. EN AW-7075 alloy, 3. weld, 4. pitting corrosion.

**Figure 10:**
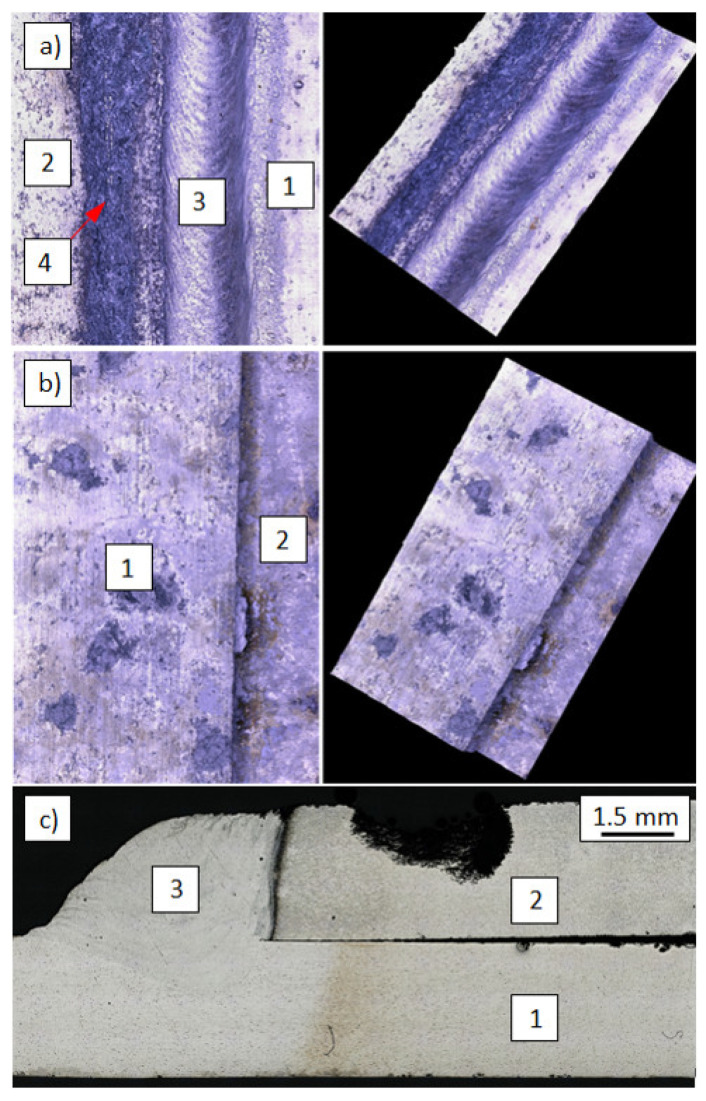
Lap welded joint after the corrosion SWAAT test where the EN AW-7075 alloy was stacked on the top-view of weld face side (**a**), back side (**b**), and cross section (**c**): 1. EN AW-6082 alloy, 2. EN AW-7075 alloy, 3. weld, 4. pitting corrosion.

Corrosion tests were carried out according to the ASTM G85 standard, using the Corrosionbox 400e (Cofomegra, Milano, Italy). The investigations confirmed corrosion resistance reduction of the EN AW-7075 in comparison to the EN AW-6082 alloy. Moreover, a significant degradation of the EN AW-7075 alloy in HAZ was revealed in an area where structural changes occurred as a result of welding heat cycles. However, it did not occur in the area of the biggest changes in the structure right next to the weld—in the fusion line (HZ), but began at a distance of approx. 1.0 mm from the weld, in the transition zone between HAZ and the base material (EN AW-7075 alloy). The figure below (Figure 9a,b) shows a part of a welded joint scanned on a light microscope, but the whole welded joint looked the same. In the case of a lap joint where the EN AW-7075 alloy is located at the bottom of the joint, pitting corrosion at the HAZ from the EN AW-7075 alloy side is visible from the side of the weld (see Figure 9a). The pitting width along the entire joint length ranges from 2.5–3.8 mm, and the depth is 0.7–1.4 mm. On the opposite side of the lap joint, in the EN AW-7075 sheet, two lines are visible along which pitting corrosion runs (Figure 10b). It penetrates the HAZ on both sides of the joint, and the width and depth of the pits are very similar to that visible from the side of the joint. On the side of the impact of the heat source, pitting corrosion occurs on both sides of the EN AW-7075 sheet, causing its degradation over almost the entire cross-section (Figure 10c). On the side of the overlap, it occurs only on one side of the EN AW-7075 sheet (from the bottom), directly exposed to the SWAAT solution. Measurements were made using the software tools available in the Keyence VHX1000 light scanning microscope (Keyence, Osaka, Japan). They enable the performance of standard measurements of geometric quantities on a 2D and 3D image.

In the second lap joint made for the reverse configuration of aluminum sheets, where the EN AW-7075 alloy was melted from the front surface, the corrosion mechanism is slightly different. From the EN AW-7075 side, the pitting corrosion is also visible running along the weld (Figure 10a). The dimensions of the resulting pitting are similar to those described above. On the other side of the joint, there are no visible pits along the HAZ (Figure 10b). No visible linear pitting corrosion on the other side of the EN AW-7075 sheet is caused by the contact of metal with the SWAAT solution limited by an overlap. In the butt joint, the situation was analogous to the visible pitting corrosion running along the joint on both sides of the weld, both from the face and the root.

In addition to the deep pitting occurring in all the welded samples, which ran along the weld, on the EN AW-7075 aluminum alloy side, the surface of the entire sheet showed spot pitting (see Figure 10b). The diameter of the pits was from 2 to 6 mm and their depth did not exceed 0.1 mm. As indicated in [25], a similar point pitting occurring on the surface of the EN AW-7075 alloy and welds was observed in friction stir welded (FSW) joints. However, no deep pits were found in the HAZ. The samples were immersed in a 3.5% NaCl solution for 60 days. Moreover, in [26], it was also shown that in the case of hybrid laser-MIG welded joints of EN AW-6061/EN AW-7075 alloys, HAZ is the most susceptible to corrosion.

**Figure 11:**
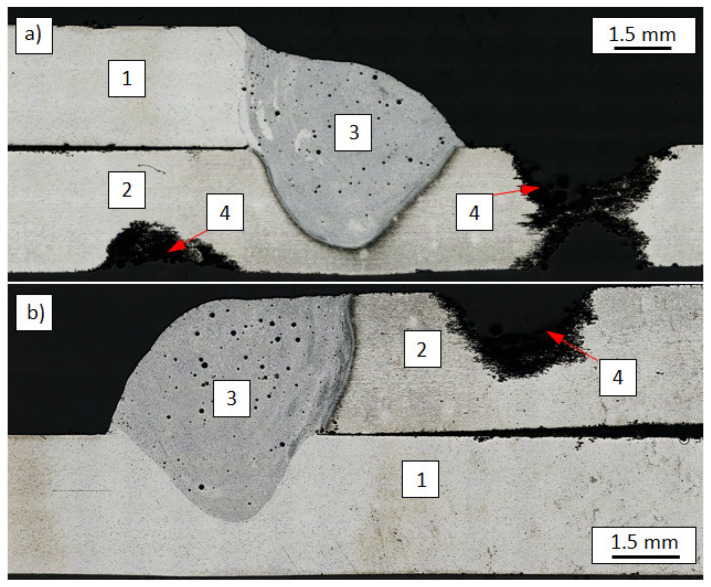
Lap welded joint after the corrosion SWAAT test made by the use of S Al 4047 filler metal. Joint where the EN AW-7075 alloy was stacked on the bottom (**a**) and reverse configuration of joint (**b**): 1. EN AW-6082 alloy, 2. EN AW-7075 alloy, 3. weld, 4. pitting corrosion.

The same corrosion progress mechanism occurs in joints made with the use of S Al 4047 filler metal (Figure 11)—additional joints, made as described in [14]. The joint made with the use of S Al 4047 filler metal has a much higher porosity than that made with the use of S Al 5183 filler metal. However, this does not reduce its corrosion resistance. Therefore, the most susceptible to the corrosion zone in the welded joints of EN AW-7075 aluminum sheets is the transition zone between the base material and the HAZ.

**Figure 12:**
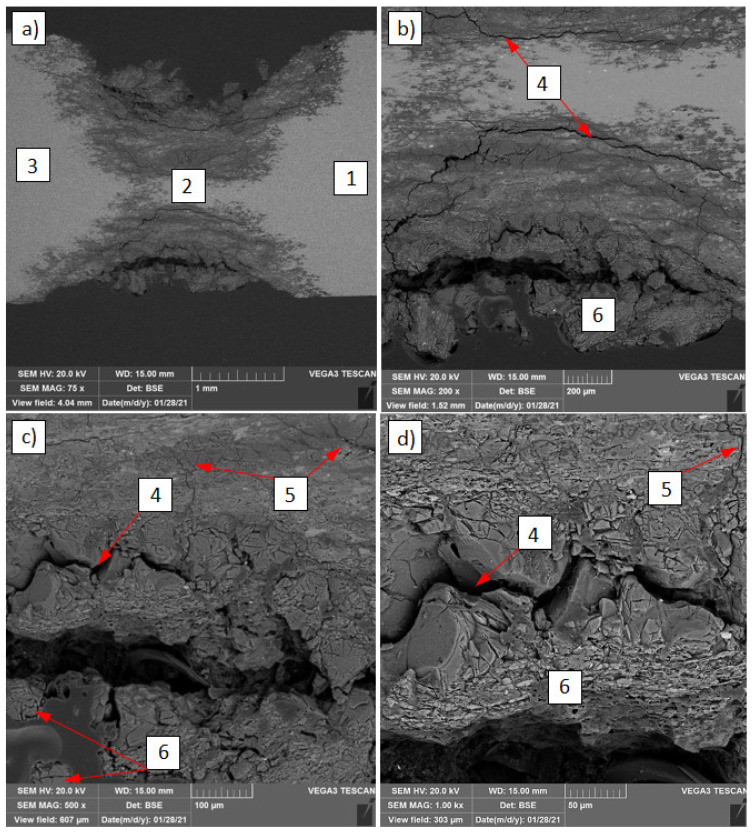
Part of lap joint after the corrosion SWAAT test made by the use of S Al 5183 filler metal from the EN AW-7075 alloy side (**a**) with different magnifications (**b**–**d**): 1. EN AW-7075 alloy, 2. transition zone between the base material EN AW-7075 alloy and the HAZ, 3. HAZ, 4. big cracks, 5. small cracks, 6. detached metal particles.

Based on the SEM analysis, it is possible to determine the mechanism of corrosion spread in the transition zone between the base material EN AW-7075 alloy and the HAZ. In those zones where corrosion is most intense, cracks along the grain boundaries are visible (Figure 12). Pitting corrosion spreads from the edge of the sheet and penetrates deep into the material. It runs along the grain boundary causing large metal grains to detach from the EN AW-7075 alloy (Figure 12a). Larger cracks allow the SWAAT solution to penetrate more easily into the material, initiating new, smaller cracks that also spread across the grain boundaries towards both the base material (Figure 12b) and the HAZ (Figure 12c,d).

Based on the energy dispersive spectrometry (EDS) point analysis performed in individual zones around the pitting corrosion, some changes in the chemical composition of the main alloying elements can be noticed (Table 4). In the transition zone between the EN AW-7075 alloy and HAZ, in which extensive pitting corrosion has been initiated, the content of Zn and Mg is reduced. The Zn content decreases to an average value of 3.1 from 7.1 wt % and Mg to 1.6 from 2.9 wt % compared to the base material (EN AW-7075 alloy). In HAZ, these changes are insignificant and close to the values measured in the EN AW-7075 alloy. Hatamleha et al. [25] has shown that pits may initiate near Al-Cu-Fe-Zn particles, which act cathodic on the 7075 alloy matrix or on Al-Mg-Zn particles, which act as anodic in relation to the matrix, thereby preferentially corroding in a salty solution. The decrease in the content of Zn and Mg elements could, therefore, change the structural composition of these compounds in the transition zone, thus significantly reducing its corrosion resistance.

**Figure 13:**
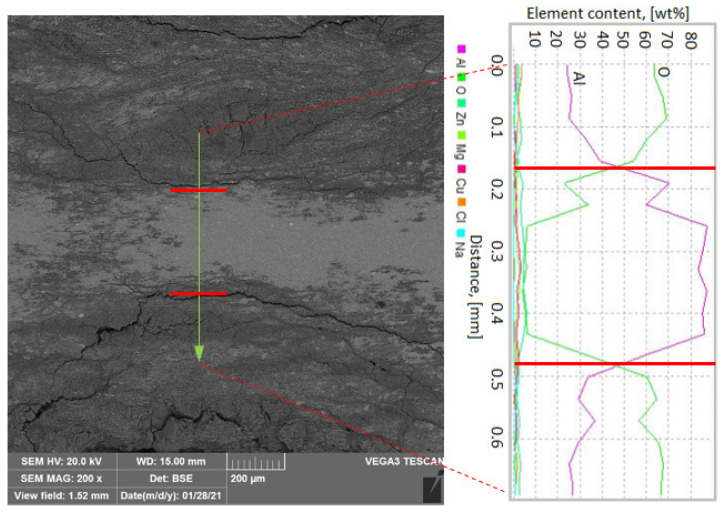
The energy dispersive spectrometry analysis performed in the transition zone between the EN AW-7075 and HAZ alloy after the corrosion tests.

The energy dispersive spectrometry (EDS) linear analysis performed in the transition zone between the EN AW-7075 and HAZ alloy (Figure 12) shows the high metal oxidation above the fracture line spreading along the grain boundary (shown in red in Figure 13). The remainder of the metal, 0.4 mm wide (from 3.0 mm), would be completely degraded quickly due to the prolonged interaction with the SWAAT solution (20 days).

## 4. Conclusions

The CMT arc welding of EN AW-7075 and EN AW-6082 aluminum sheet lap joints using the S Al 5183 filler metal is only justified when the EN AW-7075 alloy is melted from the front surface. In the case of fillet welds, where none are melted from the front surface, hot cracks occur in the joints due to the increased degree of metal mixing. The lap joints, in which this alloy is melted from the front surface, have a 38% higher shear strength (from 110 to over 150 MPa) compared to the joints made with the use of S Al 4047 filler metal. The tensile strength of the dissimilar butt joints of the alloys EN AW-7075 and EN AW-6082 is higher than the strength of the base material EN AW-6082 alloy and is on average 258 MPa. For homogeneous joints of EN AW-7075 alloy, a tensile strength of over 375 MPa can be achieved using the S Al 5183 filler metal.

The biggest changes in hardness occurred on the side of the EN AW-7075 alloy, from an average of 138 to 112 HV 0.05 in HAZ and 98 HV 0.05 in HZ and were caused by changes in the structure in these zones.

The structural changes occurring in the HAZ from the EN AW-7075 alloy, caused by the welding heat cycles, also drastically reduce the corrosion resistance in this zone. Surface pitting occurs on the entire surface of the EN AW-7075 sheet, but the most susceptible to corrosion is HAZ at a distance of approx. 1.0 mm from the weld. In this zone, corrosion spreads along the grain boundary, causing cracks and detachment of large parts of the metal from the EN AW-7075 alloy. The cracks facilitate the penetration of the SWAAT solution, initiating new, smaller cracks which also spread along the grain boundary deep into parts of the material.

## Figures and Tables

**Table 1:** Chemical composition of base material and filler metal.

Aluminum Alloy	Chemical Composition, wt %								
	Si	Fe	Cu	Mn	Mg	Cr	Zn	Ti	Al
EN AW-6082	0.70–1.30	0.45–0.55	0.08–0.12	0.40–1.00	0.60–1.20	0.23–0.27	0.18–0.22	0.08–0.12	Balance
Analysis	1.21	0.46	0.11	0.55	1.17	0.22	0.21	0.12	Balance
EN AW-7075	max 0.40	max 0.50	1.20–2.00	max 0.30	2.10–2.90	0.18–0.28	5.10–6.10	max 0.20	Balance
Analysis	0.32	0.39	1.25	0.25	2.52	0.20	5.91	0.17	Balance
S Al 5183	0.20–0.30	max 0.20	max 0.10	0.70–0.80	max 4.5	max 0.10	-	max 0.10	Balance

**Table 2:** The results of static shear (lap joints) and tensile (butt joints) tests of welded joints.

Type of Joint	Force [N]	Shear/Tensile Strength [MPa]	Average Strength [MPa]	Standard Deviation [MPa]
Lap joints with EN AW-7075 alloy in the up position	11,950	159.3	152.6	4.9
	11,250	150.0		
	11,150	148.7		
	11,700	156.0		
	11,000	146.7		
	11,600	154.7		
Lap joint with EN AW-7075 alloy in reverse configuration	7900	105.3	105.1	4.6
	7300	97.3		
	8000	106.7		
	8100	108.0		
	7700	102.7		
	8300	110.6		
Butt joints of EN AW-7075 alloy	28,750	383.3	375.9	6.0
	27,800	370.7		
	27,700	369.3		
	28,600	381.3		
	28,400	378.7		
	27,900	372.0		

**Table 3:** The results of Vickers microhardness measurements in each zone of the welded joints.

Type of Joint	Zone	Microhardness [HV 0.05]					Average Microhardness [HV 0.05]	Standard Deviation [HV 0.05]
Lap joints with EN AW-7075 alloy in the up position	BS (EN AW-6082)	80.1	86.3	85.8	80.5	84.8	83.5	3.0
	HAZ (EN AW-6082)	78.8	77.5	84.4	76.9	82.5	80.0	3.2
	Weld	101.4	109.7	107.5	109.8	103.4	106.4	3.8
	HAZ (EN AW-7075)	98.4	99.6	112.4	119.8	124.5	110.9	11.7
	BS (EN AW-7075)	137.1	141.4	139.8	141.2	137.6	139.4	2.0
Lap joint with EN AW-7075 alloy in reverse configuration	BS (EN AW-6082)	84.2	80.3	82.8	78.9	84.7	82.1	2.5
	HAZ (EN AW-6082)	80.9	82.6	79.9	82.7	79.6	81.2	1.5
	Weld	104.6	108.3	106.7	103.9	111.2	106.9	2.9
	HAZ (EN AW-7075)	101.3	106.9	115.8	120.1	127.9	114.4	10.5
	BS (EN AW-7075)	140.5	137.2	138.1	137.5	139.6	138.5	1.4
Butt joints of EN AW-7075 alloy	BS (EN AW-7075)	138.8	136.4	141.2	137.8	138.1	138.4	1.7
	HAZ (EN AW-7075)	130.5	124.6	116.5	108.9	101.3	116.36	11.7
	Weld	102.1	105.3	101.5	107.2	103.6	103.94	2.3
	HAZ (EN AW-7075)	99.5	102.3	111.8	118.3	125.4	111.46	10.8
	BS (EN AW-7075)	140.1	138.7	135.7	137.5	136.2	137.6	1.8

BS: Base material; HAZ: Heat affected zone.

**Table 4:** Results of energy dispersive spectrometry (EDS) analysis on the transition zone between EN AW-7075 and HAZ in welded lap joint after the corrosion tests.

Zone of Measurement	Chemical Composition, wt %					
	Fe	Cu	Mg	Zn	O	Al
EN AW-7075 alloy	0.3	1.9	3.2	7.1	1.1	Balance
Transition zone	0.4	1.4	1.6	3.1	2.7	Balance
Heat affected zone	0.4	1.6	2.0	5.8	1.3	Balance

## Data Availability

Data sharing is not applicable to this article.
